# Clinical Quiz: Reconstructive Strategies in a 5-Month-Old Infant with a Cloaca

**DOI:** 10.1055/s-0040-1713136

**Published:** 2020-07-12

**Authors:** Victoria A. Lane, Marc A. Levitt

**Affiliations:** 1 Department of Paediatric Surgery, Royal Victoria Infirmary, Newcastle upon Tyne, United Kingdom of Great Britain and Northern Ireland; 2 Department of Surgery, Center for Colorectal and Pelvic Reconstructive Surgery, Children's National Hospital, District of Columbia, Washington, United States

**Keywords:** cloaca, cloacagram, anorectal malformation

## Abstract

Herein we present a case of 5-month-old female born with a cloaca. She underwent a colostomy at birth and then underwent endoscopy and cloacagram to plan for the definitive reconstruction. The case is presented with a focus on the reconstructive strategies, and questions for the readers are posed in a quiz format.

## Introduction

You are in the preoperative planning stage for a 5-month-old female with a known cloaca. She has been defunctioned in the newborn period with a colostomy, but did not require a vaginostomy or intermittent catheterization for hydrocolpos.

**Fig. 1:**
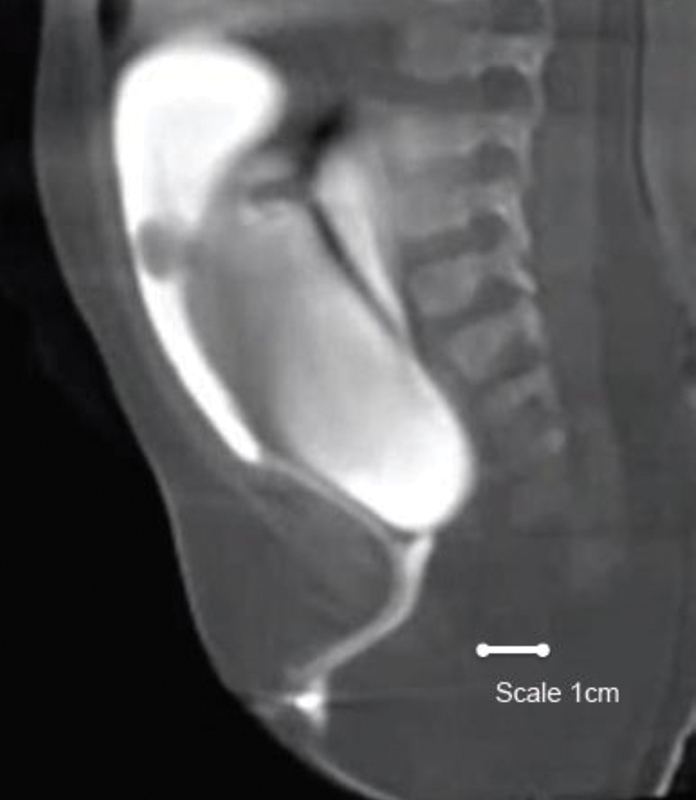
Cloacagram (using water soluble contrast).

The radiologist has performed a cloacagram study as demonstrated in Fig. 1 .

Questions are as follows:

1. Would you consider this a minor or a complex type of cloaca?
  - Minor cloaca
  - Complex cloaca
2. What is the length of the common channel (CC)?
  - 1 cm
  - 2 cm
  - 3 cm
  - 4 cm
3. What is the length of the urethra—the distance from the CC to the bladder neck?
  - 1 cm
  - 1.5 cm
  - 2 cm
  - 2.5 cm
  - 3 cm
  - 4 cm
4. What would be your surgical approach for the reconstruction?
  - Posterior sagittal incision only and total urogenital mobilization (TUM).
  - Posterior sagittal incision plus laparotomy/laparoscopy and transabdominal TUM.
  - Posterior sagittal incision only and urogenital separation. (i.e., separate the vagina and rectum from the CC and leave CC to become the urethra).
  - Posterior sagittal incision and laparotomy/laparoscopy with urogenital separation. (i.e., separate the vagina and rectum from the CC and leave CC to become the urethra).

Answers are as follows:

Question 1: b, complex cloaca.
Question 2: c, the CC is 3 cm.
Question 3: d, the urethral length is 2.5 cm.
Question 4: d, a urogenital separation is best given by the long CC and desire not to disrupt the urogenital diaphragm which would occur if a TUM was performed.

## Discussion

In this case, the CC is 3-cm long. The urethra is 2.5-cm long. A standard approach would advise that a <3-cm CC can potentially be managed with a TUM 1 2 ; however, in this case, there are additional factors to take into consideration. 1

Based on the cloacagram 3 performed in addition to the endoscopy, the reconstructive plan becomes clear.

The urethra, measured from its take off from the CC to the bladder neck is long (> 1.5 cm) which in general points to a situation amenable to a TUM. 1 4 However, in this case, both the urethral length and the length of the CC must simultaneously be considered. If one was to mobilize the urogenital complex by doing a TUM, the CC would be split to allow the urethral orifice to reach the perineum. Getting the urethral orifice to the perineum would require pulling down the urogenital complex, which risks disruption of the function of the bladder neck. Such a significant mobilization would move the bladder neck below the urogenital diaphragm and urinary leakage and incontinence may result.

It is therefore advised that the CC plus the length of the urethra to be used as the final urethral length, 1 5 and this can be achieved by performing a urogenital separation. The vagina is removed from the back of the CC and it is repaired, thus the entire CC plus urethra becomes the patient's urethra, and the bladder neck remains in its original position.

**Fig. 2:**
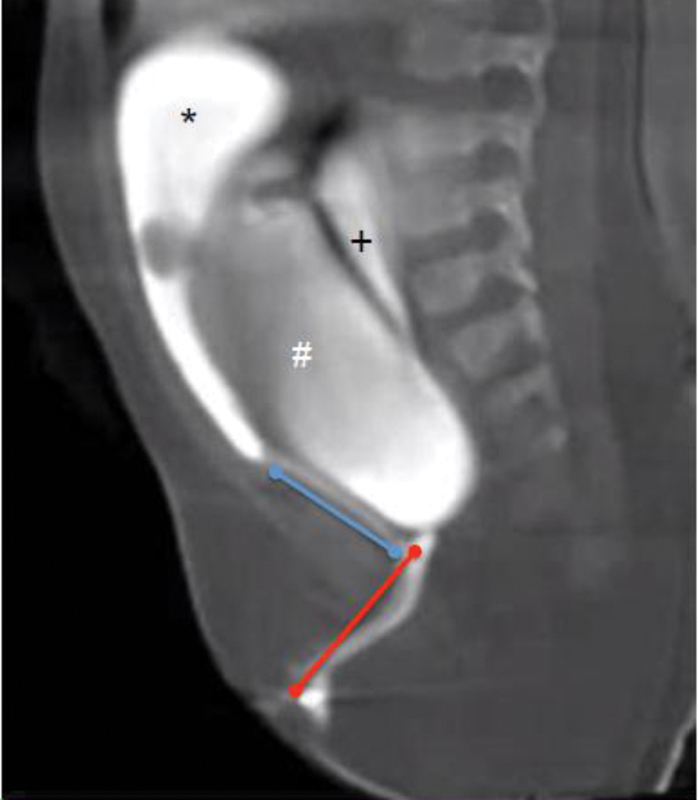
Common channel: 3 cm (red line); Urethra: 2.5 cm (blue line). (*) Bladder, (#) Vagina, (+) Rectum.

If a surgeon was to embark on a TUM and the urogenital complex did not reach the perineum, which would likely be the case here, then they would need to perform a transabdominal TUM, meaning that the dissection would need to be continued via the abdomen. The problem with this approach is that if this maneuver does not allow the urethra and introitus to reach the perineum, then at that point a urogenital separation would be required, and this is a problematic situation. Remember that, an TUM requires dissection of the anterior urethra to mobilize the urogenital sinus en bloc. This approach works well for most short cloacae (<3 cm). 4 If the surgeon starts an TUM and then realizes intraoperatively that the urogenital structures will not reach the perineum, the only option at that point is to convert to a urogenital separation. This would create a situation whereby the entire circumference of the urethra has already been dissected, and is a situation which could render the urethra ischemic, leading to a high chance of urethral loss ( Fig. 2 ).

The second important reconstructive planning to consider is that this case will require a laparotomy/laparoscopy to identify and mobilize the rectum. The rectum is extremely high (behind the sacrum) and will not be reached using a posterior sagittal incision alone.

## Conclusion

Therefore, given these considerations, we suggest beginning with a posterior sagittal approach and starting the urogenital separation. Then the abdomen is entered to mobilize the rectum, completing the urogenital separation, and finally pulling through the native vagina and the rectum.

## Notes

New Insights and the Importance for the Pediatric Surgeon Educational article to highlight the importance of preoperative planning in complex cloacal malformations.
